# Impact of Genetic Testing on the Diagnosis, Management, and Prognosis of Hypertrophic Cardiomyopathy: A Systematic Review

**DOI:** 10.7759/cureus.70993

**Published:** 2024-10-07

**Authors:** Billy McBenedict, Wilhelmina N Hauwanga, Emmanuel S Amadi, Aaron A Abraham, Rithika Sivakumar, Madeleine O Okere, Melvin Chun Yang Yau, Nematalla Balla, Thasneem Rahumathulla, Berley Alphonse, Bruno Lima Pessôa

**Affiliations:** 1 Neurosurgery, Fluminense Federal University, Niterói, BRA; 2 Family Medicine, Faculty of Medicine, Federal University of the State of Rio de Janeiro, Rio de Janeiro, BRA; 3 Internal Medicine, Hallel Hospital Port Harcourt, Port Harcourt, NGA; 4 Internal Medicine, Christian Medical College Ludhiana, Ludhiana, IND; 5 College of Medicine, Government Medical College, Omandurar Government Estate, Chennai, IND; 6 Internal Medicine, University of Port Harcourt Teaching Hospital, Port Harcourt, NGA; 7 College of Medicine, Monash University Malaysia, Subang Jaya, MYS; 8 Internal Medicine, National Ribat University, Doha, QAT; 9 Medicine and Surgery, Tbilisi State Medical University, Tbilisi, GEO; 10 Internal Medicine, University Notre Dame of Haiti, Port-au-Prince, HTI

**Keywords:** autosomal dominant, diagnosis and management, genes, genetic testing genes, hypertrophic cardiomyopathy (hcm)

## Abstract

Hypertrophic cardiomyopathy (HCM) is a hereditary cardiovascular condition marked by heart muscle thickening, fibrosis, and myocardial disorders. It is often inherited in an autosomal dominant pattern. Symptoms include dyspnea, fatigue, palpitations, dizziness, syncope, and an increased risk of sudden cardiac death (SCD). Genetic studies have identified many asymptomatic carriers, indicating a higher prevalence of HCM. Advances in genetic testing (GT) and novel therapies, such as cardiac myosin inhibitors, have significantly impacted the diagnosis and management of HCM. This integrative review followed the Preferred Reporting Items for Systematic Reviews and Meta-Analyses (PRISMA) guidelines and aimed to synthesize information regarding the impact of GT on the diagnosis and management of HCM patients. An electronic search was conducted on May 17, 2024, across PubMed, Embase, Scopus, Web of Science, and Cochrane databases, covering January 2020 to May 2024. Inclusion criteria were studies involving adult HCM patients who underwent GT and follow-up. Exclusion criteria included non-human studies, pediatric cases, non-HCM-related GT, non-peer-reviewed articles, systematic reviews, conference abstracts, and editorials. From 1,155 articles identified, 42 met the inclusion criteria after applying filters and removing duplicates. GT identified pathogenic variants in a significant proportion of HCM patients, enhancing diagnostic accuracy and management. Key mutations were found in myosin binding protein C3 and myosin heavy chain 7 genes. GT facilitated personalized management strategies, including risk stratification for SCD and family screening. Patients with identified mutations often required closer monitoring and tailored treatments. GT has revolutionized the diagnosis and management of HCM. The integration of genetic data has improved risk stratification, facilitated early intervention, and enhanced patient outcomes. Despite these advances, challenges remain in identifying genetic variants in some patients, emphasizing the need for continuous improvement in genetic panels and diagnostic methods. This review highlights the significant role of GT in optimizing HCM care through precise risk assessment and tailored treatment strategies.

## Introduction and background

Hypertrophic cardiomyopathy (HCM) is a common hereditary cardiovascular condition characterized by thickening of the heart muscle, increased fibrosis, and myocardial disorders. It affects approximately one in 500 adults, or 0.2% of the general population, and is predominantly inherited in an autosomal dominant manner, with environmental influences also playing a significant role [1,2].

Symptoms of HCM vary and include dyspnea, fatigue, palpitations, dizziness, syncope, atypical chest pain, and an elevated risk of sudden cardiac death (SCD) [3]. The severity of these symptoms is not always correlated with the degree of heart muscle thickening or ventricular obstruction, highlighting the complexity of the disease. HCM is a leading cause of SCD in young adults, especially athletes, due to its impact on cardiac function and the propensity for serious arrhythmias such as atrial fibrillation and ventricular tachycardia, which also increase the risk of stroke [4].

Genetic studies in families with HCM and the general population have identified mutation carriers who show no phenotypic expression (i.e., at the preclinical stage) or have an unknown clinical status, suggesting a higher prevalence of HCM than previously estimated [1]. HCM is commonly caused by mutations in cardiac sarcomere genes such as myosin heavy chain 7 (MYH7) and myosin binding protein C3, but a significant proportion of cases have no identifiable mutations [5]. This lack of identifiable mutations contributes to the underestimation of the disease's prevalence, leaving many asymptomatic carriers undiagnosed [1].

Novel targeted pharmacotherapies, specifically cardiac myosin inhibitors, have shown promise in reversing key pathophysiological changes and altering the disease course of HCM. The favorable outcomes associated with these therapies led to the early approval of mavacamten by the Food and Drug Administration, marking a significant shift in HCM treatment [6]. In addition, treatment options include implantable defibrillators, symptom-relieving medications, and procedures such as surgical myectomy or alcohol septal ablation to reduce left ventricular outflow obstruction. Despite these advancements, many patients continue to face significant challenges in managing HCM, including variability in disease progression, adverse effects of medications, limited access to advanced therapies, and the need for ongoing monitoring and lifestyle adjustments [6].

Advances in molecular genetics and sequencing technologies, such as next-generation sequencing, have revolutionized the diagnosis of HCM. These technologies allow for more accurate identification of pathogenic variants and facilitate family tracking of asymptomatic carriers who may require regular monitoring. Studies indicate that mutations in the sarcomere, especially in genes such as MYH7, are associated with more severe forms of the disease and a greater risk of adverse cardiovascular events [5]. Despite these advances, challenges remain in identifying genetic variants in patients without known mutations in typical HCM genes, highlighting the continued need to improve genetic panels and diagnostic methods [7].

Genetic testing revolutionizes the diagnosis and treatment of HCM, providing accurate information about its pathogenesis and guiding personalized therapies to improve clinical outcomes. This integrative review aims to analyze the impact of these tests on clinical practice, highlighting their influence on therapeutic decisions and healthcare and identifying areas for future research and development of more effective clinical guidelines.

## Review

Materials and methods

The systematic review followed the Preferred Reporting Items for Systematic Reviews and Meta-Analyses (PRISMA) guidelines for organizing and reporting its results [8]. An electronic search was conducted across several research databases, including PubMed, Embase, Scopus, Web of Science, and Cochrane. The search strategy and the filters used are indicated in Table 1. All databases were accessed on May 17, 2024, and the search covered the period from January 2020 to May 2024.

**Table 1:** Summary of the search strategy from the databases

Database	Search strategy	Filters used
PubMed	(("testing genetic"[Title/Abstract] OR "testing genetic predisposition"[Title/Abstract] OR "Genetic Testing"[Title/Abstract]) AND ("Hypertrophic Cardiomyopathies"[Title/Abstract] OR "Hypertrophic Cardiomyopathy"[Title/Abstract] OR "cardiomyopathy hypertrophic obstructive"[Title/Abstract] OR "Hypertrophic Obstructive Cardiomyopathies"[Title/Abstract] OR "Hypertrophic Obstructive CardiomyopathY"[Title/Abstract] OR "obstructive cardiomyopathy hypertrophic"[Title/Abstract]))	Humans only, English language, exclude preprints, filter years 2020-2024
Embase	('testing, genetic':ab,ti OR 'testing, genetic predisposition':ab,ti OR 'genetic testing':ab,ti) AND ('hypertrophic cardiomyopathies':ab,ti OR 'hypertrophic cardiomyopathy':ab,ti OR 'cardiomyopathy, hypertrophic obstructive':ab,ti OR 'cardiomyopathies, hypertrophic obstructive':ab,ti OR 'hypertrophic obstructive cardiomyopathies':ab,ti OR 'hypertrophic obstructive cardiomyopathy':ab,ti OR 'obstructive cardiomyopathies, hypertrophic':ab,ti OR 'obstructive cardiomyopathy, hypertrophic':ab,ti)	Humans only, English language, filter years 2020-2024
Scopus	(TITLE-ABS-KEY ("Testing, Genetic" OR "Testing, Genetic Predisposition" OR "Genetic Testing") AND TITLE-ABS-KEY ("Hypertrophic Cardiomyopathies" OR "Hypertrophic Cardiomyopathy" OR "Cardiomyopathy, Hypertrophic Obstructive" OR "Cardiomyopathies, Hypertrophic Obstructive" OR "Hypertrophic Obstructive Cardiomyopathies" OR "Hypertrophic Obstructive Cardiomyopathy" OR "Obstructive Cardiomyopathies, Hypertrophic" OR "Obstructive Cardiomyopathy, Hypertrophic"))	Humans only, English language, filter years 2020-2024
Web of Science	“Testing, Genetic” OR “Testing, Genetic Predisposition” OR “Genetic Testing” (Abstract) and “Hypertrophic Cardiomyopathies” OR “Hypertrophic Cardiomyopathy” OR “Cardiomyopathy, Hypertrophic Obstructive” OR “Cardiomyopathies, Hypertrophic Obstructive” OR “Hypertrophic Obstructive Cardiomyopathies” OR “Hypertrophic Obstructive CardiomyopathY” OR “Obstructive Cardiomyopathies, Hypertrophic” OR “Obstructive Cardiomyopathy, Hypertrophic” (Abstract)	Humans only, English language, filter years 2020-2024
Cochrane	(“Testing, Genetic” OR “Testing, Genetic Predisposition” OR “Genetic Testing” AND “Hypertrophic Cardiomyopathies” OR “Hypertrophic Cardiomyopathy” OR “Cardiomyopathy, Hypertrophic Obstructive” OR “Cardiomyopathies, Hypertrophic Obstructive” OR “Hypertrophic Obstructive Cardiomyopathies” OR “Hypertrophic Obstructive CardiomyopathY” OR “Obstructive Cardiomyopathies, Hypertrophic” OR “Obstructive Cardiomyopathy, Hypertrophic”):ti,ab,kw	Humans only, English language, filter years 2020-2024

Inclusion and Exclusion Criteria

The inclusion criteria encompassed studies involving human subjects and adults diagnosed with HCM on whom genetic testing (GT) (e.g., sequencing) was performed and patient follow-up (followed or monitored for a period of time). Eligible study designs included primary research studies published in English. Clinical cases were included only if they involved more than 10 participants. Studies reporting on the patient follow-up (followed or monitored for a period of time) on whom GT (e.g., sequencing) was performed were of interest. The systematic review included various studies examining the role of GT in HCM across diverse populations (Table 2). Only peer-reviewed journal articles in English were considered for inclusion. Exclusion criteria encompassed non-human, pediatric or not related to GT of HCM patients, non-peer-reviewed articles, systematic reviews or reviews, conference abstracts, and editorials.

**Table 2:** Studies that were used to synthesize this review, with their respective demographics and key results MYBPC3 = myosin binding protein C3; MYH7 = myosin heavy chain 7; GT = Genetic testing; HCM = hypertrophic cardiomyopathy; HF = heart failure; SARC+ = sarcomere gene mutations; CPET = cardiopulmonary exercise testing; G+ = genotype-positive; LP/P = likely pathogenic or pathogenic; ECG = electrocardiogram; CMR = cardiac magnetic resonance; TTE = transthoracic echocardiography; DNA = deoxyribonucleic acid; LVH = left ventricular hypertrophy; ICD = implantable cardioverter-defibrillator; SCD = sudden cardiac death; PLN = phospholamban

Author	Type of study	Key findings
Zhou et al. [9]	The study included 392 participants with a mean age of 46.4 years; 68.37% were male​.	GT in HCM identified key gene mutations MYBPC3 and MYH7, enhancing diagnostic accuracy and enabling personalized treatment for patients with distinct hypertrophic patterns.
Zhou et al. [10]	The study included 198 HCM patients, 48% men, with an average age of 47 ± 13 years​.	GT in HCM, revealing MYBPC3 and MYH7 mutations, significantly enhances diagnostic accuracy and management, especially with younger patients and those with a family history.
Zhang et al. [11]	The study involved 893 HCM patients, with 66.1% men, and a mean age of 47.2 ± 14.2 years​.	GT in HCM identified MYBPC3 mutations, highlighting the link between right ventricular involvement and increased risk of cardiovascular and HF-related deaths, guiding targeted management.
Wu et al. [12]	The study included 1,000 HCM patients, 64.5% men, with a median age of 47.9 ± 14.6 years​.	GT for FHOD3 variants in HCM revealed increased cardiovascular and all-cause death risks, emphasizing its importance in clinical risk stratification and management.
Wasserstrum et al. [13]	The study included 1,328 HCM patients, 34% women, with a median age of 56 years​.	GT in hypokinetic HCM identified MYBPC3 and MYH7 variants, highlighting earlier disease onset and progression, underscoring its importance in early diagnosis and personalized management.
Velicki et al. [14]	The study included 63 HCM patients, 24% women, with a mean age of 51.1 ± 14.2 years.	GT in HCM identified MYBPC3 and MYH7 mutations, with MYH7 linked to more severe clinical phenotypes, emphasizing its role in risk stratification and personalized management.
Trachoo et al. [15]	70.97% were men, with a mean age of 53.03 ± 15.87 years.	GT in HCM identified MYBPC3 variants and emphasized cardiovascular surveillance, and cascade testing, enabling early diagnosis and targeted management to prevent morbidity and mortality.
Stava et al. [16]	4408 cardiomyopathy probands (65.1% male, mean age 50.9 years) and 3008 relatives (47.6% male, mean age 39.3 years).	GT in HCM identified pathogenic variants in 11.9% of cases, aiding early intervention and tailored treatment, while family testing tripled the identification of at-risk relatives.
Ramensky et al. [17]	1,685 individuals, with 1,056 females (median age 52) and 629 males (median age 44).	GT in HCM identified MYBPC3 and MYH7 pathogenic variants, guiding close monitoring and early interventions, thus improving patient management and clinical outcomes.
Nakashima et al. [18]	203 patients (130 men, average age 61.8 years) were analyzed for SARC+ and clinical characteristics​.	GT in HCM revealed SARC+ in 33% of patients, who were younger, had severe clinical features, and higher risks of morbid events, guiding management strategies.
Morita et al. [19]	99 patients; 45% had a positive genotype. The average age was 40±17 years for positive genotype and 55±22 years for the negative genotype; 38% and 33% were female.	GT in HCM found pathogenic variants in 26% of patients, with higher yields in those with higher genotype predictor scores, enhancing management strategies.
Moriki et al. [20]	209 patients, 70% were men, with an average age of 56 ± 16 years​.	GT in HCM identified pathogenic variants in 26% of patients, with higher prevalence in familial cases, guiding personalized management through a validated genetic test prediction score.
​Meshkov et al. [21]​	214 participants, 48.6% were men, with a median age of 35.5 years.	GT in HCM identified pathogenic variants in 43.8% of patients, linked to earlier onset and severe disease, guiding personalized management and improving patient outcomes.
McGurk et al. [22]	214 participants, 48.6% male, 29.9% children, with a median age of 35.5 years​.	GT in HCM identified pathogenic variants in 41% of patients, enabling precise risk stratification and tailored management, crucial for improving patient outcomes.
Mazzaccara et al. [23]	133 patients, 60.2% were male, with a median age of 36 years​.	GT in HCM identified pathogenic variants in 40% of patients, aiding accurate diagnosis, risk stratification, and tailored management, especially for those with uncommon gene variants.
Mattivi et al. [24]	121 Mayo Clinic and 90 Australian patients, 50% were male with a mean age of 34 years​.	GT in HCM identified pathogenic variants in 41% of patients, significantly enhancing risk stratification and management, particularly for those at higher risk of HF and SCD.
Magrì et al. [25]	371 patients, predominantly male (64%), with a mean age of 49 ± 16 years​.	GT in HCM identified pathogenic variants in 55% of patients, linked to earlier onset and higher HF risk, though CPET-derived clinical variables were stronger outcome predictors.
Lorenzini et al. [26]	285 individuals (49.5% male) with LP/P variants; the median age at evaluation was 14.2 years.	GT in HCM revealed a 46% 15-year penetrance, with higher rates in males, emphasizing the importance of regular ECG monitoring and mutation-specific management strategies.
Lakdawala et al. [27]	214 participants, 48.6% male, 29.9% children, with a median age of 35.5 years​.	GT in HCM identified sarcomere mutations in 46.1% of patients, with later diagnoses in females, guiding personalized management and improving clinical outcomes.
Lacaze et al. [28]	13,131 participants, 54% were female, with a mean age of 75 years​.	GT in asymptomatic older adults identified pathogenic variants in 0.2%, influencing clinical decisions and highlighting the low penetrance of HCM over 4.7 years.
Huurman et al. [29]	214 participants, 48.6% were men, and the median age was 35.5 years.	GT, combined with CMR imaging, reclassified 27% of G+ individuals with normal ECG and TTE as having HCM, optimizing early detection and management.
Huurman et al. [30]	91 subjects, 40% were male, with a mean age of 46 ± 13 years​.	GT identified sarcomere variants in non-hypertrophic individuals, with reduced left atrial strain predicting G+ status but not HCM progression, highlighting the need for additional predictive markers.
Hughes et al. [31]	50 G+, LVH-negative subjects (17 men, mean age 37.6 years) and 28 age- and sex-matched healthy volunteers (14 men, mean age 37.9 years).	GT identified sarcomeric gene variants in first-degree relatives of HCM patients, revealing subclinical abnormalities via CMR, emphasizing the importance of early intervention before significant hypertrophy develops.
Huang et al. [32]	227 patients with HCM were analyzed, with a mean age of 43 years, including 152 men.	GT identified SARC+ in 47.1% of HCM patients, correlating with increased myocardial fibrosis and poorer outcomes, enhancing risk stratification and personalized management.
Holliday et al. [33]	3 unrelated patients (2 females, aged 65 and 56, 1 male, aged 48).	GT using transcriptome sequencing identified novel MYBPC3 splice-altering variants in HCM patients, enabling tailored therapeutic interventions, including antisense oligonucleotide treatment, in cases where traditional DNA sequencing was inconclusive.
Heliö et al. [34]	3208 adult cardiomyopathy patients (mean age 50 years, 63.6% male) from 69 centers in 18 countries.	GT in HCM identified pathogenic variants in 43.3% of patients, aiding risk stratification and personalized management, including increased use of implantable cardioverter defibrillators.
​Hathaway et al. [35]​	1376 HCM patients (64.2% male, 35.7% female) with an average age of 44.7 years​.	GT in HCM identified pathogenic variants in 26.8% of patients, primarily in sarcomeric genes, facilitating precise management and informing family members about potential risks.
Earle et al. [36]	336 HCM probands (36% women, 64% men) with a mean age of 46 years​.	GT in HCM identified pathogenic variants in 40% of patients, enabling precise risk stratification and personalized management, with MYBPC3 and MYH7 being the most affected.
de la Rosa et al. [37]	78 adults, with 39 G+ HCM patients (35.9% women, mean age 51.9 years) and 39 age- and gender-matched hypertensive LVH patients (35.9% women, mean age 54.8 years).	GT in HCM revealed more advanced diastolic dysfunction and greater LVH compared to hypertensive LVH, guiding tailored management strategies.
de Feria et al. [38]	127 HCM probands (64% male, median age 32.5 years for sarcomere positive, 51 years for sarcomere negative)​.	GT in HCM identified sarcomere mutations in 54% of patients, enabling tailored management, with mutation carriers showing a younger diagnosis age and higher family history prevalence.
Dai et al. [39]	793 HCM patients (mean age 51 years, 69.4% male) and 414 healthy controls.	GT identified RBM20 variants in 2.4% of HCM patients, linked to higher sudden cardiac arrest prevalence, improving risk stratification and personalized management.
Chumakova et al. [40]	The study included 193 HCM patients (52% male, median age 56 years) from a Russian single-center cohort​.	GT in Russian HCM patients identified causative variants in 38%, enhancing risk stratification and management, with thin filament variant carriers showing a worse HF prognosis.
Chen et al. [41]	178 HCM patients (56.2% male, mean age 55.2 years) across three cohorts.	GT in HCM identified 45% of patients as G+, correlating with younger age, severe dysfunction, and higher SCD risk; a deep learning ECG model improved risk stratification.
Canepa et al. [42]	7286 HCM patients (59.5% male, mean age 48 years) from the SHaRe registry​.	GT in HCM identified pathogenic variants in 45.1% of patients, improving risk stratification and management, with decreased yield over time reflecting older, milder cases.
Butzner et al. [43]	The study included 1,841 patients with obstructive HCM (52% male, mean age 63.2 years)​.	GT in HCM identified pathogenic variants in 38.7% of patients, enhancing risk stratification and management, with MYBPC3 and MYH7 being the most prevalent, leading to targeted interventions like defibrillator implantation.
Butters et al. [44]	836 HCM probands (64.9% male, mean age 54.9 years).	GT in HCM identified pathogenic variants in 39.3% of patients, aiding risk stratification and personalized management, with significant ethnic disparities in testing uptake and ICD implantation.
Boen et al. [45]	99 heart transplant recipients with prior non-ischemic cardiomyopathy (mean age 47.8 years, 22.2% female)​.	GT in heart transplant recipients with non-ischemic cardiomyopathy identified pathogenic variants in 38.7% of patients, particularly TTN variants, facilitating targeted interventions and family screening.
Arabadjian et al. [46]	434 HCM patients (41.5% female, 13.1% Black, mean age 54.6 years for Black patients, 62.5 years for White patients)​.	GT in HCM identified pathogenic variants in 43.5% of Black patients and 30.3% of White patients, guiding personalized treatment and highlighting higher risks in Black patients.
Antoniutti et al. [47]	59 patients from eight families (49% female, median age 39 years).	GT identified pathogenic mutations in 40% of HCM patients, enabling targeted management and risk stratification, particularly for MYBPC3 mutation carriers, and facilitated early diagnosis in family members.
Amr et al. [48]	283 patients were included, with 67% male and a mean age of 50 years.	GT identified disease-causing variants in 49% of HCM patients, significantly improving SCD risk stratification and predictive accuracy and enhancing management strategies to prevent SCD.
Allouba et al. [49]	514 patients (67% male, mean age 34.7 years) and 400 controls​.	In an Egyptian cohort, GT increased the yield of clinically actionable variants from 19% to 29.6%, with a higher prevalence of homozygous variants, improving diagnosis and personalized management of HCM.
Afana et al. [50]	16 patients (62.5% female, median age 51 years).	GT identified the PLN p.Leu39* variant in 4.8% of HCM patients, facilitating personalized monitoring for arrhythmias and family screening, leading to better risk stratification and management.

Results

**Figure 1:**
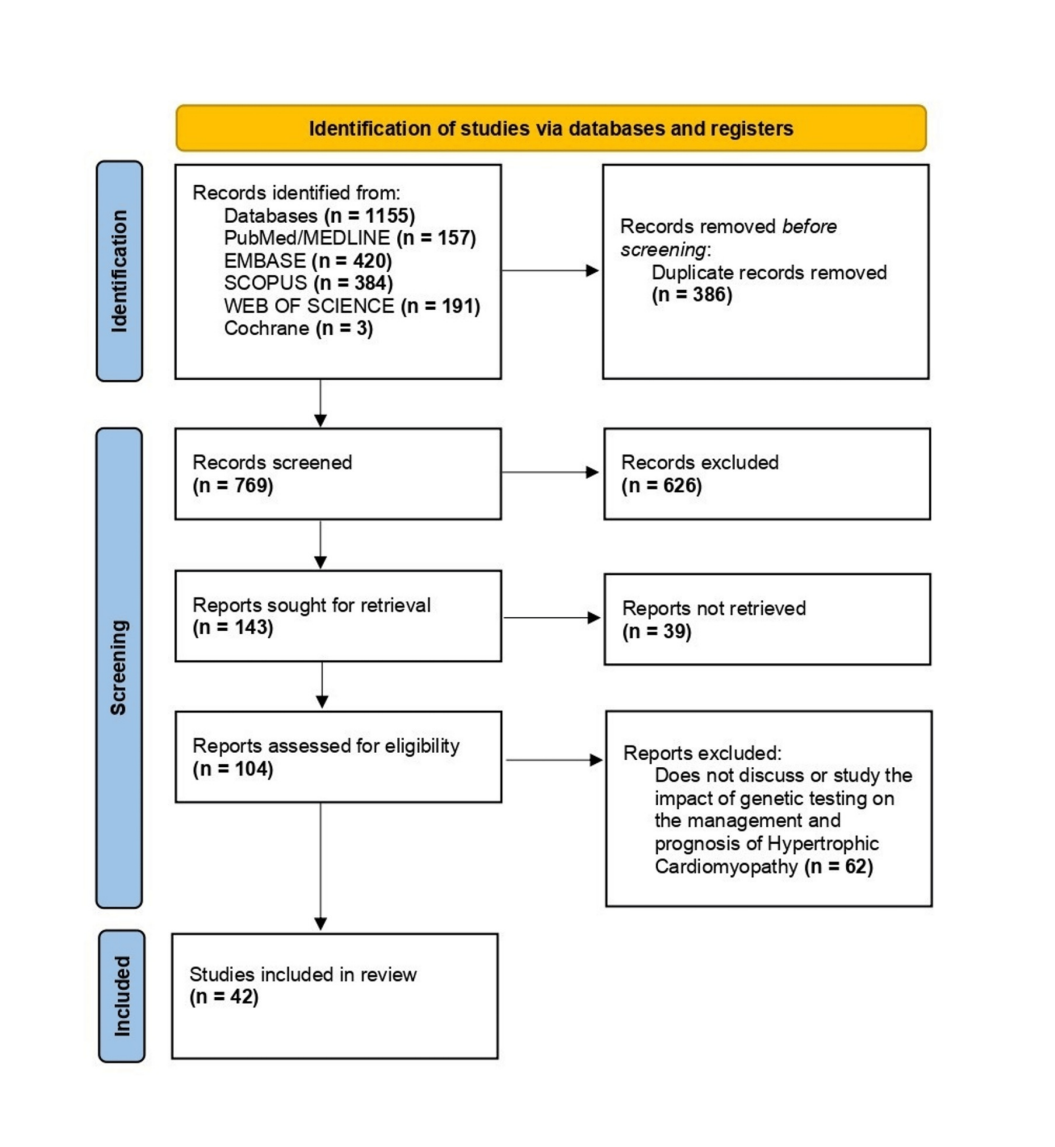
Preferred Reporting Items for Systematic Reviews and Meta-Analyses (PRISMA) flow diagram indicating the steps taken to filter the articles for this review

Through our search strategy, we identified a total of 1,155 articles (Figure 1), comprising 157 from PubMed/Medline, 420 from Embase, 384 from Scopus, 191 from Web of Science, and three from Cochrane. Filters were applied based on the inclusion/exclusion criteria. The articles were transferred to an Excel sheet, where 386 duplicates were manually removed, resulting in 769 articles. These 769 articles were further scrutinized based on their titles and abstracts, leading to the disqualification of 626, leaving 143 articles. Full texts for 39 articles could not be retrieved, leaving us with 104 papers for eligibility assessment. After a thorough full-text review, 62 papers were excluded, resulting in 42 articles being included in the final review (Table 2). Data screening was conducted by two authors that worked independently, with a third reviewer consulted in cases of disagreement. Notably, no automated tools were utilized in this process. The systematic review included various studies examining the role of GT in HCM across diverse populations (Table 2). More information can be obtained in the appendices.

Study Quality and Bias Assessment

The Joanna Briggs Institute (JBI) Critical Appraisal Checklist outlines several common issues that can impact the validity and reliability of articles included in a systematic review. These issues include selection bias due to non-comparable groups, measurement bias from invalid or unreliable assessments of exposure and outcomes, and confounding from unidentified or poorly controlled confounding factors. Other concerns include attrition bias from loss to follow-up, inappropriate statistical analysis, and reporting bias due to selective or non-transparent result reporting. Addressing these challenges is essential to ensure accurate and reliable findings in cohort studies, facilitating their use in clinical practice and further research. The quality of the 41 articles was evaluated using the JBI Critical Appraisal Tools (Appendix A). All studies included in the analysis focused on a clearly defined issue regarding the impact of GT on the diagnosis, management, and prognosis of HCM. Each study recruited participants appropriately, clearly stating inclusion criteria and participant demographic data (Appendix B). However, many studies showed significant gender representation differences, with a particularly low proportion of male participants compared to females, complicating meaningful comparisons.

Variations in the number of participants involved in each study can cause bias in a systematic review by affecting the conclusions drawn. Studies with larger sample sizes generally provide more reliable and generalizable results due to the reduced impact of random variation. Conversely, studies with smaller sample sizes may produce less reliable outcomes, potentially skewing the overall findings of the review. This disparity can lead to an overestimation or underestimation of the effectiveness of an intervention, ultimately affecting the conclusions made about its efficacy and safety.

Variations in follow-up methods, such as the use of both clinical and telephonic follow-ups as seen in Wu et al.'s [12] study, can also influence the outcomes. Different follow-up methods may yield varying levels of detail and accuracy in the data collected. For instance, clinic follow-ups might provide more thorough assessments compared to telephonic follow-ups, which might miss subtle clinical changes. This inconsistency in follow-up approaches can introduce bias, as the outcomes may appear better or worse depending on the method used, thereby impacting the reliability of the conclusions in the systematic review.

Discussion

GT plays a crucial role in the diagnosis and management of HCM by identifying pathogenic variants that may inform clinical decisions. A study identified the phospholamban (PLN) p.Leu39* variant in 4.8% of HCM patients and 3.2% of dilated cardiomyopathy (DCM) patients [50]. The detection of this variant facilitated targeted clinical interventions, such as personalized monitoring for arrhythmias and family screening. Patients with the PLN variant exhibited higher incidences of non-sustained ventricular arrhythmias compared to those with myosin-binding protein C3 (MYBPC3)/myosin heavy chain 7 (MYH7)-related cardiomyopathies. The presence of pathological Q waves and elevated N-terminal pro-B-type natriuretic peptide levels (median value 4893.2 ± 6299.5 pg/dL) were more frequent among PLN carriers, indicating a distinct clinical profile ​[50]​. This genetic information allowed for more accurate risk stratification and tailored management strategies, ultimately improving patient outcomes ​[50]​.

GT has played a pivotal role in the management of HCM in an Egyptian cohort study. The study revealed that incorporating ancestry-matched controls increased the yield of clinically actionable variants from 19% to 29.6%, demonstrating the importance of GT in identifying pathogenic variants. Notably, the prevalence of homozygous variants was significantly higher in Egyptian patients (4.1%) compared to UK patients (0.1%), and these variants were more likely to occur in minor HCM genes like MYL2, MYL3, and CSRP3, which were less penetrant in heterozygosity but contributed to biallelic disease in consanguineous populations ​[49]​. This enhanced understanding of genetic variants facilitated more accurate diagnoses and personalized management strategies, thereby improving patient outcomes ​[49]​. In another study with a cohort of 283 HCM patients, GT identified disease-causing variants in 49% of the cases [48]​. Patients with a positive genotype had a significantly higher risk of SCD, with a hazard ratio (HR) of 3.13 compared to genotype-negative patients. The integration of genetic findings into the existing European Society of Cardiology (ESC) five-year SCD risk model increased the model's predictive accuracy, with the area under the curve (AUC) improving from 0.735 to 0.76. This enhanced risk model demonstrated a sensitivity of 85.7% and a specificity of 69.1% at a 6% risk threshold, compared to the original model's sensitivity of 28.6% and specificity of 83.3%. The modified model reduced the number needed to treat for preventing one SCD event to 9, compared to 13 for the ESC model and 28 for the American Heart Association/American College of Cardiology guidelines. These findings underscore the importance of GT in accurately identifying high-risk patients and tailoring management strategies to prevent SCD​ [48]​.

GT played a pivotal role in the diagnosis and management of HCM by identifying pathogenic mutations in a significant proportion of HCM patients, with a diagnostic yield of approximately 40%. This precise genetic information allowed for targeted management strategies, including risk stratification for SCD and tailored therapeutic interventions. For instance, patients with mutations in the MYBPC3 gene, which accounted for 20% of the diagnosed cases, were monitored more closely due to their higher risk of adverse events. Furthermore, family members of mutation carriers benefited from predictive testing, facilitating early diagnosis and preventive measures ​[47]​. Another study used GT on 45.1% of Black patients and 43.1% of White patients, revealing likely pathogenic or pathogenic (LP/P) variants in 43.5% of Black patients compared to 30.3% of White patients. This genetic information enabled precise risk stratification and personalized treatment strategies. For instance, Black patients were found to have a higher prevalence of massive left ventricular hypertrophy (LVH) (38.5% vs. 6.9%) and multiple sudden death risk factors (61.5% vs. 23.6%), necessitating more aggressive monitoring and intervention. The identification of genetic variants also informed family screening and guided clinical decision-making, enhancing overall patient outcomes ​[46]​.

GT played a pivotal role in the diagnosis and management of HCM by significantly improving diagnostic yield and guiding patient management in a study with 31 heart transplant recipients with non-ischemic cardiomyopathy. It revealed an LP/P variant in 38.7% of patients. Specifically, truncating variants in the TTN gene were the most prevalent, found in 24.1% of DCM cases. In addition, the diagnostic yield was similar among patients with and without a family history of cardiomyopathy, highlighting the broad applicability of GT. This genetic information facilitated targeted clinical interventions, including family screening and personalized management plans. Through cascade screening, 39.6% of family members were found to carry a pathogenic variant, with 52.6% of these carriers exhibiting a cardiac phenotype, thereby enabling early diagnosis and intervention to prevent disease progression and improve clinical outcomes ​[45]. 

A study included 836 unrelated HCM probands and found that 82.4% of patients underwent GT, with 39.3% receiving an LP/P result. This genetic information enabled more precise risk stratification and personalized management strategies, such as the identification of MYBPC3 and MYH7 gene variants, which accounted for 59% and 27.9% of LP/P cases, respectively. In addition, the uptake of GT varied significantly between ethnic groups, with only 57.3% of East Asian patients undergoing GT compared to 86.4% of Europeans. The study also highlighted disparities in implantable cardioverter-defibrillator (ICD) implantation, with East Asian patients having significantly lower rates (19%) compared to other ethnic groups (30-36%). These findings underscore the importance of GT in tailoring management plans and improving clinical outcomes for HCM patients [44].

In the study, 38.7% of patients who underwent GT were found to carry LP/P variants. Among these, variants in the MYBPC3 and MYH7 genes were the most prevalent, detected in 59% and 27.9% of cases, respectively. This genetic information allowed for better risk stratification and personalized management strategies. For example, the identification of specific genetic variants informed the decision to implant cardioverter-defibrillators, which were used in 8% of patients to prevent SCD. Moreover, GT facilitated family screening and early diagnosis of at-risk relatives, further improving overall patient outcomes and guiding appropriate therapeutic interventions​ [43]. In another study, GT was performed in 61.7% of the 7,286 patients, revealing LP/P variants in 45.1% of those tested. The yield of GT decreased over time, from 57.7% before 2000 to 38.4% after 2010, reflecting a shift toward diagnosing older patients with milder phenotypes and sporadic disease. This genetic information has allowed for better risk stratification and personalized management strategies, including family screening and early intervention for at-risk relatives. The prevalence of obstructive HCM increased in recent cohorts, with peak gradients over 30 mm Hg rising from 31.9% before 2000 to 39.0% after 2010, highlighting the evolving clinical landscape of HCM management influenced by genetic insights ​[42]​.

In a study, 45% of the 178 HCM patients tested were found to be genotype-positive (G+). Patients who were G+ were younger, had more severe cardiac dysfunction, and had a higher likelihood of having a family history of SCD (17.5% vs. 4.08%, p < 0.01). The deep learning-derived 12-lead electrocardiogram (ECG) model outperformed traditional methods, achieving an AUC of 0.89 compared to 0.69 for both the Mayo and Toronto scores (p < 0.01). This model also demonstrated higher sensitivity (0.84) and positive predictive value (0.87) than the conventional scoring systems. By identifying G+ patients more accurately, the model facilitated better risk stratification and tailored management plans, including the consideration of ICDs to prevent SCD​ [41]​. Another study involving 193 Russian HCM patients reported that GT identified 64 causative variants in 66 patients (38%). The recurrent variants included MYBPC3 p.Q1233* (found in eight patients), MYBPC3 p.R346H (2), MYH7 p.A729P (2), TPM1 p.Q210R (3), and FLNC p.H1834Y (2). The presence of these genetic variants facilitated more precise risk stratification and personalized management strategies. For instance, carriers of thin filament variants (such as TPM1) had a worse prognosis for heart failure (HF) (HR = 7.9, p = 0.007), emphasizing the need for targeted clinical interventions. Genetic findings also led to family screening, identifying additional affected relatives and enabling early diagnosis and intervention to prevent disease progression and improve clinical outcomes​ [40].

GT significantly impacted the diagnosis and management of HCM by identifying the RBM20 gene as a potential causal factor [39]. In the study, 793 patients with HCM and 414 healthy controls underwent exome sequencing, revealing 14 rare deleterious variants in the RBM20 gene, with 2.4% of HCM patients carrying these variants compared to 0.72% of controls. Patients with RBM20 variants exhibited a higher prevalence of sudden cardiac arrest (6.7% vs. 0.9%, p = 0.001) and increased SCD risk factor counts. These genetic insights allowed for better risk stratification and personalized management strategies, including enhanced surveillance for malignant arrhythmias and consideration of preventive measures for high-risk patients ​[39]​. GT significantly influenced the management of HCM by identifying the presence or absence of sarcomere gene mutations (SARC+). In the study of 127 HCM patients, 54% tested positive for pathogenic sarcomere mutations [38]. Those with sarcomere mutations were over three times more likely to have a family history of HCM (66% vs. 17%) and were diagnosed at a younger age (median age 32 vs. 51 years, p < 0.0001). Conversely, patients without sarcomere mutations were significantly more obese (body surface area p = 0.003, body mass index p = 0.04) and more likely to present with left ventricular (LV) outflow tract obstruction (54% vs. 35%, p = 0.0483). This genetic information enabled tailored management strategies, such as early intervention for younger patients with a family history of HCM and targeted lifestyle modifications for older, obese patients​ [38]​.

In a study comparing 39 patients with gene-positive HCM (G+/P+) to 39 patients with hypertensive LVH, the G+/P+ group showed more advanced diastolic dysfunction, larger left atrial size (22.1 cm² vs. 18.9 cm², P = 0.002), and a higher incidence of moderate diastolic dysfunction (33.3% vs. 12.8%, P = 0.032). Furthermore, the G+/P+ group exhibited greater LV mass (277.7 g vs. 207.7 g, P = 0.025) and more severe LVH (58.8% vs. 27.3%, P = 0.047). These findings highlight the importance of GT in identifying patients with gene-positive HCM, which allows for more tailored management strategies to address the increased risk of HF and other complications ​[37]​. In a study of 336 HCM probands, 40% were found to carry P/LP variants, with the MYBPC3 and MYH7 genes being the most commonly affected, accounting for 60% and 24% of cases, respectively [36]. Patients with P/LP variants were diagnosed at a younger age (mean age 39 ± 17 years) compared to those without such variants (mean age 51 ± 17 years, P < 0.001), and were more likely to experience severe clinical events, such as cardiac arrest or SCD (P = 0.002). This genetic information enabled more precise risk stratification and personalized management strategies, including enhanced monitoring and tailored interventions for high-risk patients. Furthermore, cascade genetic screening in variant-positive families identified additional at-risk relatives, facilitating early diagnosis and preventative care​ [36]​. In another study involving 1,376 patients with suspected HCM, GT identified LP/P variants in 26.8% (369/1,376) of cases [35]. The majority of these diagnostic variants (85%) were found in genes encoding sarcomeric proteins, crucial for cardiac muscle function. Factors such as earlier age at diagnosis, higher maximum wall thickness, positive family history, absence of hypertension, and presence of an ICD were associated with higher diagnostic yield. These genetic insights facilitated more precise patient management and informed family members about potential risks, leading to targeted surveillance and therapeutic strategies ​[35]​.

In a study of 3208 adult cardiomyopathy patients, GT was performed in 48.8% of HCM cases, revealing at least one disease-causing variant in 43.3% of the HCM patients. Patients with GT were younger at diagnosis (median age 45 vs. 50 years, p < 0.001), more likely to have familial disease (60% vs. 22%, p < 0.001), and a family history of SCD (21.7% vs. 10.8%, p < 0.001). This genetic information facilitated more precise risk stratification and personalized management strategies, including the use of implantable cardioverter defibrillators, which were more frequently implanted in genetically tested patients (30.5% vs. 20.6%, p < 0.001) [34]​. GT significantly influenced HCM patient management by identifying novel splice-altering variants in the MYBPC3 gene [33]. 

In a study involving transcriptome sequencing of patient-specific induced pluripotent stem cell-derived cardiomyocytes (hiPSC-CMs), researchers identified pathogenic splicing in two individuals with known MYBPC3 splice-gain variants and discovered cryptic exon splicing due to an MYBPC3 c.1928-569G>T variant in a patient with an unresolved genetic cause [33]. This novel approach facilitated the genetic diagnosis of HCM in cases where traditional deoxyribonucleic acid (DNA) sequencing was inconclusive, enabling tailored therapeutic interventions. In addition, antisense oligonucleotide treatment demonstrated significant inhibition of cryptic exon splicing in patient-specific hiPSC-CMs, highlighting a potential personalized therapeutic option for managing HCM​ [33]. In a study involving 227 patients with HCM who underwent GT and cardiac magnetic resonance (CMR) imaging, 107 patients (47.1%) were found to carry SARC+ ​[32]​. These patients exhibited a higher myocardial fibrosis ratio both in histopathology (15.3% vs. 12.4%, p = 0.003) and in CMR imaging (98.1% late gadolinium enhancement (LGE) positive vs. 84.2%, P<0.001; LGE quantification 8.3% vs. 5.8%, p < 0.001). The study found a significant correlation between SARC+ and increased myocardial fibrosis, which is associated with poorer clinical outcomes. This genetic information facilitated more precise risk stratification and personalized management strategies, emphasizing the importance of GT in HCM management ​[32]​.

GT was used to identify LP/P variants in sarcomeric protein genes among first-degree relatives of individuals diagnosed with HCM ​[31]. This led to the recruitment of 50 G+, LVH-negative subjects. CMR revealed that these subjects had longer indexed anterior mitral valve leaflets (12.52 ± 2.1 vs. 11.55 ± 1.6 mm/m², p = 0.03), lower LV end-systolic volume (21.0 ± 6.9 vs. 26.7 ± 6.2 mm/m², p ≤ 0.005), and higher LV ejection fraction (71.9 ± 5.5 vs. 65.8 ± 4.4%, p ≤ 0.005) ​[31]. Visual perfusion defects were observed in 20% of mutation carriers compared to none in the control group (p = 0.011). These findings underscored the importance of GT in identifying subclinical HCM, facilitating early intervention and management adjustments before significant hypertrophy or scarring occurs ​[31]. This corroborates with another study that identified pathogenic DNA variants in family members of affected patients [29]; of 91 G+ individuals with a maximal wall thickness (MWT) less than 15 mm, 27% were reclassified as having HCM based on CMR imaging, despite normal findings on ECG and transthoracic echocardiography (TTE). Specifically, 34% of subjects with no HCM on TTE were diagnosed with HCM on CMR. These reclassifications were predominantly due to CMR's ability to detect myocardial thickening and other subtle abnormalities not visible on TTE, such as anterobasal hooks ​[29]. Subjects with normal ECG and TTE results were rarely reclassified, justifying the use of these initial screening tools before resorting to CMR. This approach helped to optimize clinical management and resource allocation, underscoring the value of GT combined with advanced imaging techniques in the early detection and intervention of HCM ​[29]​.

GT was also used to identify sarcomere gene variants in individuals who did not yet exhibit hypertrophy [30]. This testing helped classify a G+, phenotype-negative (Ph−) group. Among the 160 screened, 91 subjects were included in the study. Logistic regression analysis revealed that left atrial strain (LASr) was significantly reduced in G+/Ph− subjects compared to healthy controls (32.7% vs. 36.7%, p = 0.002) ​[30]​. LASr, along with other echocardiographic parameters such as MWT and mitral inflow velocities, independently predicted G+ status. However, LASr did not predict the progression to HCM during a median follow-up of 5.9 years, with 25% of the G+/Ph− subjects eventually developing HCM. These findings indicate that while GT effectively identifies at-risk individuals, additional markers are necessary to predict disease progression and optimize management strategies ​[30]​.

Among the 13,131 asymptomatic older adults tested in a study, 24 individuals (0.2%) were identified as carriers of pathogenic variants associated with HCM [28]. These findings influenced clinical decisions, highlighting the potential need for preventive measures. Despite the detection of these variants, none of the carriers experienced SCD during the mean follow-up period of 4.7 years, indicating a low penetrance of the disease in this population. This study underscores the role of GT in identifying at-risk individuals and guiding clinical management, even in asymptomatic older adults [28]​. In a cohort of 3,788 patients who underwent GT, 46.1% were found to have SARC+, with a higher prevalence in females (50.9%) compared to males (43.3%) ​[27]. This genetic information influenced the management strategies, as females with SARC+ were diagnosed approximately 3.6 years later than their male counterparts, indicating potential delays in disease onset or recognition ​[27]​. Furthermore, the study highlighted that women with specific gene mutations, such as MYBPC3 and thin filament variants, were diagnosed at older ages compared to men (4.8 and 6.7 years later, respectively), suggesting a difference in disease penetrance and progression based on genetic factors​ [27]. This genetic stratification facilitated more personalized management approaches, including timely interventions and monitoring for those at higher risk, thereby enhancing clinical outcomes and providing a deeper understanding of HCM's genetic basis.

In a cohort of 285 sarcomere protein mutation carriers who did not initially fulfill diagnostic criteria for HCM, 86 patients (30.2%) developed HCM over a median follow-up of eight years [26]. The study found that the 15-year penetrance of HCM was 46%, with male carriers exhibiting a higher penetrance (58%) compared to female carriers (33%) [26]​. An abnormal ECG was a strong predictor of HCM development, with an adjusted HR of 4.02, highlighting the importance of regular ECG monitoring ​[26]​. Genetic stratification based on specific mutations, such as MYBPC3 and MYH7, informed personalized management strategies, with TNNI3 mutations showing the lowest risk (HR of 0.19)​ [26]. These findings underscore the critical role of GT in early detection, risk stratification, and tailored long-term surveillance in managing HCM.

GT was used to identify LP/P variants in 55% of the 371 patients studied [25]. These genetic findings were associated with a more aggressive disease phenotype, including an earlier age of onset (45 ± 16 years for those with LP/P variants versus 53 ± 18 years for those without, p < 0.001) and a higher risk of HF events ​[25]​. Despite this, multivariate analysis indicated that clinical variables derived from cardiopulmonary exercise testing, such as left atrial diameter, circulatory power percentage, and ventilatory efficiency versus carbon dioxide production slope, were stronger predictors of HF and SCD than the presence of LP/P variants ​[25]​. Similarly, a study identified LP/P variants in 41% of the 665 patients studied [24]. This genetic insight was pivotal as LP/P variant carriers had a markedly higher risk of adverse outcomes, including SCD and HF events. Specifically, LP/P variant carriers exhibited a higher incidence of HF-related events (23% vs. 15%, p = 0.02) and a non-significant trend toward increased SCD (7% vs. 3%, p = 0.09) compared to non-carriers [24]​. The incorporation of genetic data into patient management allowed for more precise risk stratification, enhancing clinical decision-making and personalized care​ [24]​.

In a cohort of 133 patients, genetic analysis revealed that 40% carried at least one P/LP variant, with 28% of these variants found in uncommon genes [23]. This genetic information has proven critical for accurate diagnosis and risk stratification. Notably, patients with HCM carrying P/LP variants in uncommon genes exhibited specific clinical features, such as asymmetric LVH and exertional dyspnea. This detailed genetic profiling allows for tailored management plans, improving patient outcomes by addressing individual genetic risks and manifestations​ [23]​. In another cohort study involving 10,400 individuals referred for HCM genetic panel sequencing, 41% were found to carry rare, protein-altering variants in well-established disease-associated genes [22]. The presence of these genetic variants allowed for precise risk stratification, which was crucial in managing patient outcomes. The study also highlighted that pathogenic variants had an estimated penetrance of 22.5%, while likely pathogenic variants had a penetrance of 10.7%. This genetic information guided clinicians in tailoring management plans, emphasizing the importance of GT in both diagnosis and long-term management strategies for HCM patients​ [22].

Furthermore, a study involving 665 patients used GT to identify LP/P variants in 43.8% of the participants, with the most frequently mutated genes being MYBPC3 and MYH7 [21]. The presence of these genetic variants was associated with earlier onset and more severe disease manifestations, including an increased risk of adverse cardiac events. Specifically, LP/P variant carriers had a higher incidence of HF and a trend towards increased SCD [21]​. This genetic information was crucial for risk stratification, informing decisions on interventions such as the implantation of cardioverter defibrillators and the initiation of more aggressive medical therapy. Overall, the integration of GT into the clinical management of HCM has enabled more precise and personalized treatment approaches, improving patient outcomes [21]. 

In a study involving 209 Japanese HCM patients, 26% were found to have LP/P variants, with a higher prevalence in familial cases (60%) [20]. The Mayo Clinic phenotype-based genetic test prediction score, validated in this cohort, showed a strong correlation between higher scores and positive genetic test results, with yields ranging from 8% for patients with a score of -1 to 100% for those with a score of 4. This score facilitates targeted GT, enabling personalized management plans for patients and their families based on genetic risk factors​ [20]. In a study evaluating the Mayo Clinic HCM genotype predictor score among 209 Japanese patients, 26% were found to have LP/P variants, with a higher prevalence (60%) in familial cases. The study demonstrated that patients with higher genotype predictor scores had increased yields of positive genetic tests, ranging from 8% in those with a score of -1 to 100% in those with a score of 4. This correlation suggests that the genetic test prediction score is a valuable tool for anticipating the likelihood of detecting genetic mutations in HCM, thereby guiding more precise and effective management strategies ​[19]​.

In a study involving 203 Japanese patients with HCM, genetic analyses revealed that 33% had SARC+ [18]. Patients with these mutations were significantly younger at diagnosis and exhibited more severe clinical features, such as increased interventricular wall thickness and higher incidences of non-sustained ventricular tachycardia [18]. Importantly, mutation-positive patients experienced significantly more HCM-related morbid events, particularly lethal arrhythmic events, throughout their lifetimes compared to those without mutations [18]. The study underscored the importance of genetic information in predicting complications and guiding management strategies, highlighting that patients with sarcomere mutations had an HR of 2.08 for HCM-related morbid events compared to those without mutations ​[18]​. 

GT significantly influenced the diagnosis and management of HCM in a targeted sequencing of 242 clinically important genes among 1,658 individuals from the Ivanovo region ​[17]. Pathogenic variants were identified in multiple genes associated with HCM, including MYBPC3 and MYH7. Specifically, the MYBPC3 variant (rs376395543) and the MYH7 variant (rs121913650) were identified as pathogenic, with the MYBPC3 variant observed in three individuals and the MYH7 variant in one individual ​[17]. The identification of these variants provided critical insights into disease management, such as the necessity for close monitoring and potential early interventions to prevent adverse outcomes like SCD. The study highlighted that the presence of these pathogenic variants required tailored management strategies, emphasizing the value of GT in improving clinical outcomes for HCM patients [17]​.

In a study involving 4,408 cardiomyopathy probands, 11.9% of those with HCM had an LP/P variant detected [16]. Genetic family testing identified three times as many variant-positive relatives, allowing for preventive measures. Moreover, the study revealed that GT's diagnostic yield was substantial, with sarcomeric genes such as MYBPC3 and MYH7 frequently implicated, supporting the genetic basis for targeted clinical strategies and familial screening protocols ​[16]​. In a study involving 62 subjects (31 with HCM and 31 with DCM, genetic variants were detected in 48.39% of HCM cases, with 32.26% classified as LP/P and 16.13% as variants of uncertain significance [15]. The most prevalent gene associated with HCM was MYBPC3. The study also emphasized the importance of cardiovascular surveillance and cascade GT among asymptomatic first-degree relatives, resulting in a detection rate of 8.82% for new HCM cases. This proactive genetic approach not only facilitated early diagnosis but also informed targeted management strategies, underscoring the potential to prevent morbidity and mortality in at-risk family members​ [15]​.

In a study including 63 patients with HCM, GT identified mutations in the MYBPC3 gene in 76% of cases and in the MYH7 gene in 24% of cases [14]. Patients with the MYH7 mutation exhibited a more severe clinical phenotype, characterized by a higher prevalence of atrial fibrillation (60% vs. 35%, p = 0.085) and significant mitral valve abnormalities, including systolic anterior motion (33% vs. 10%, p = 0.025) and mitral leaflet abnormalities (40% vs. 19%, p = 0.039) [14]. In addition, MYH7 mutation carriers had higher LV filling pressures (E/e′ ratio of 13.9 ± 6.9 vs. 8.8 ± 3.3, p = 0.079). These findings underscore the importance of GT in identifying high-risk patients, guiding more intensive monitoring and tailored therapeutic strategies to improve clinical outcomes [14]​. In a study of 83 patients with hypokinetic HCM, GT was conducted in 53 patients, revealing that 72% had one or more pathogenic variants [13]. The most common gene implicated was MYBPC3 (in 20 patients), followed by MYH7 (in seven patients). Patients with these pathogenic variants were diagnosed with HCM at a younger age (26 years vs. 50 years) and developed HCMr earlier (51 years vs. 67 years) [13]. The presence of multiple pathogenic variants, particularly in the MYBPC3 gene, further accelerated disease onset and progression. These genetic insights underscore the importance of GT in guiding early diagnosis, family screening, and personalized management strategies to improve outcomes in HCM patients​ [13].

In a study involving 1,000 patients with HCM and 761 controls, 27 FHOD3 candidate variants were detected in 33 patients (3.3%) compared to 12 variants in 12 controls (1.6%), highlighting a significant difference (odds ratio, 2.13; p < 0.05) [12]. The presence of FHOD3 variants was associated with an increased risk of cardiovascular death and all-cause death, with an adjusted HR of 3.71 for cardiovascular death and 3.02 for all-cause death [12]. These findings emphasize the importance of incorporating FHOD3 GT into clinical practice for better risk stratification and management of HCM patients​ [12]​.

In a study of 893 HCM patients, 669 underwent GT, revealing a higher prevalence of G+ results in patients with right ventricular (RV) involvement (57.0%) compared to those without RV involvement (31.0%, p < 0.001) [11]​. Notably, LP/P variants of the MYBPC3 gene were significantly more frequent in patients with RV involvement (30.4% vs. 12.0%, p < 0.001) [11]​. The study demonstrated that RV involvement, indicated by structural abnormalities such as RV hypertrophy, obstruction, and LGE, was an independent risk factor for cardiovascular death (adjusted HR 4.191, p = 0.002), all-cause death (adjusted HR 3.013, p = 0.011), and HF-related death (adjusted HR 14.142, p = 0.004) [11]​. These findings underscore the importance of GT in identifying high-risk HCM patients and facilitating targeted management and surveillance strategies​ [11]​. A study analyzed 198 HCM patients and found a GT yield of 49.5%, with predominant mutations in MYBPC3 (41.84%) and MYH7 (34.69%) [10]. G+ patients were younger at diagnosis (48.98% vs. 35.00% under 45 years, p = 0.046), had a higher family history of HCM (29.59% vs. 15.00%, p = 0.014), and exhibited greater LV MWT (23.70 ± 5.66 mm vs. 21.53 ± 5.56 mm, p = 0.007) [10]. In addition, the combination of a deep learning model with the Toronto genotype score significantly improved predictive performance for HCM mutations, achieving an AUC of 0.84 and 84.31% accuracy, which aids in selecting patients for cost-effective GT and informs better management strategies​ [10].

In a study involving 392 HCM-affected families, comprehensive genetic evaluation using whole-exome sequencing revealed significant correlations between genetic mutations and distinct hypertrophic patterns [9]. Specifically, mid-septal hypertrophy was predominantly associated with MYBPC3 variants, while a higher septum-to-posterior wall ratio was linked to MYH7 variants [9]. These findings facilitated more accurate identification of relevant mutations, particularly those of unknown significance, enhancing the precision of genetic diagnosis. Notably, the study reported a 49.5% overall yield of GT, with 76.5% of causative mutations detected in MYBPC3 and MYH7 [9]. This integration of genetic and echocardiographic data not only improved diagnostic accuracy but also informed personalized management plans for HCM patients, emphasizing the value of "gene-echocardiography" in clinical practice [9]​. 

## Conclusions

In studies included in this review, GT played a pivotal role in the diagnosis and management of HCM by identifying pathogenic variants that inform clinical decisions. The detection of the PLN p.Leu39* variant facilitated targeted interventions and family screening, revealing a distinct clinical profile with higher incidences of non-sustained ventricular arrhythmias and elevated N-terminal pro-B-type natriuretic peptide levels. Incorporating ancestry-matched controls in GT increased the yield of actionable variants, particularly highlighting the significance of homozygous variants in Egyptian patients. In addition, GT improved risk stratification for sudden cardiac death and enhanced the predictive accuracy of existing risk models, thereby facilitating personalized management strategies. These findings underscore the importance of GT in optimizing HCM care through precise risk assessment, early intervention, and tailored treatment plans, ultimately improving patient outcomes and guiding appropriate therapeutic interventions.

## Appendices

Appendix A

**Table 3:** Risk-of-bias assessment

	Zhou et al. [9]	Zhou et al. [10]	Zhang et al. [11]	Wu et al. [12]	Wasserstrum et al. [13]	Velicki et al. [14]	Trachoo et al. [15]	Stava et al. [16]	Ramensky et al. [17]	Nakashima et al. [18]	Morita et al. [19]	Moriki et al. [20]	​Meshkov et al. [21]​	McGurk et al. [22]	Mazzaccara et al. [23]	Mattivi et al. [24]	Magrì et al. [25]	Lorenzini et al. [26]	Lakdawala et al. [27]	Lacaze et al. [28]	Huurman et al. [29]	Huurman et al. [30]	Hughes et al. [31]	Huang et al. [32]	Holliday et al. [33]	Heliö et al. [34]	​Hathaway et al. [35]​	Earle et al. [36]	de la Rosa et al. [37]	de Feria et al. [38]	Dai et al. [39]	Chumakova et al. [40]	Chen et al. [41]	Canepa et al. [42]	Butzner et al. [43]	Butters et al. [44]	Boen et al. [45]
Were there clear criteria for inclusion in the case series?	Y	Y	Y	Y	Y	Y	Y	Y	Y	Y	Y	Y	Y	Y	Y	Y	Y	Y	Y	Y	Y		Y	Y	Y	Y	Y	Y	Y	Y	Y	Y	Y	Y	Y	Y	Y
Was the condition measured in a standard, reliable way for all participants included in the case series?	Y	Y	Y	Y	Y	Y	Y	Y	Y	Y	Y	Y	Y	Y	Y	Y	Y	Y	Y	Y	Y	Y	Y	Y	Y	Y	Y	Y	Y	Y	Y	Y	Y	Y	Y	Y	Y
Were valid methods used for the identification of the condition for all participants included in the case series?	Y	Y	Y	Y	Y	Y	Y	Y	Y	Y	Y	Y	Y	Y	Y	Y	Y	Y	Y	Y	Y	Y	Y	Y	Y	Y	Y	Y	Y	Y	Y	Y	Y	Y	Y	Y	Y
Did the case series have the consecutive inclusion of participants?	Y	Y	Y	Y	Y	Y	Y	Y	Y	Y	Y	Y	Y	Y	Y	Y	Y	Y	Y	Y	Y	Y	Y	Y	Y	Y	Y	Y	Y	Y	Y	Y	Y	Y	Y	Y	Y
Did the case series have a complete inclusion of participants?	Y	Y	Y	Y	Y	Y	Y	Y	Y	Y	Y	Y	Y	Y	Y	Y	Y	Y	Y	Y	Y	Y	Y	Y	Y	Y	Y	Y	Y	Y	Y	Y	Y	Y	Y	Y	Y
Was there clear reporting of the demographics of the participants in the study?	Y	Y	Y	Y	Y	Y	Y	Y	Y	Y	Y	Y	Y	Y	Y	Y	Y	Y	Y	Y	Y	Y	Y	Y	Y	Y	Y	Y	Y	Y	Y	Y	Y	Y	Y	Y	Y
Were the outcomes or follow-up results of cases clearly reported?	Y	Y	Y	Y	N/C	Y	N/A	Y	N/A	Y	N/C	N/C	N/C	N	N	Y	Y	Y	Y	Y	N	Y	N/C	Y	N/A	N/C	N/C	Y	Y	Y	Y	Y	Y	Y	Y	Y	Y
Was there clear reporting of the presenting sites’ or clinics’ demographic information?	Y	Y	Y	Y	Y	Y	Y	Y	Y	Y	Y	Y	Y	Y	Y	Y	Y	Y	Y	Y	Y	Y	Y	Y	Y	Y	Y	Y	Y	Y	Y	Y	Y	Y	Y	Y	Y
Was the statistical analysis adequate?	Y	Y	Y	Y	Y	Y	Y	Y	Y	Y	Y	Y	Y	Y	Y	Y	Y	Y	Y	Y	Y	Y	Y	Y	Y	Y	Y	Y	Y	Y	Y	Y	Y	Y	Y	Y	Y

Appendix B

**Table 4:** Genes linked to HCM, their respective clinical manifestations, and management. HCM: hypertrophic cardiomyopathy, MYBPC3: myosin-binding protein C, MYH7: beta-myosin heavy chain, ALPK3: alpha kinase 3, TNNI3: troponin I3, TNNT2: troponin T2, TPM1: tropomyosin 1, TTN: titin, OBSCN: obscurin, FHOD3: formin homology 2 domain-containing 3, SCN5A: sodium channel, voltage-gated, type V, alpha subunit, ICD: implantable cardioverter-defibrillator, ACE: angiotensin-converting enzyme, CRT: cardiac resynchronization therapy, LVH: left ventricular hypertrophy, DCM: dilated cardiomyopathy, SCD: sudden cardiac death, NSVT: non-sustained ventricular tachycardia, AF: atrial fibrillation, LVMWT: left ventricular maximal wall thickness, LVPWT: left ventricular posterior wall thickness, LVOT: left ventricular outflow tract, SRT: septal reduction therapy, HTx: heart transplantation, PLN: phospholamban, LVD: left ventricular dilation, HF: heart failure

Author	Genes	Clinical manifestations	Management/treatment
Zhou et al. [9] 2023	MYBPC3	Mid-septal hypertrophy.	Beta-blockers, Calcium channel blockers, or Septal myectomy
	MYH7	Higher septum-to-posterior wall ratio, left ventricular outflow tract obstruction, hypertrophy pronounced in the anterior wall, interventricular septum, and lateral wall of the left ventricle.	Beta-blockers, Calcium channel blockers, or Septal myectomy
	ALPK3	Apical hypertrophy. The study suggests that mutations in ALPK3 are linked to reduced obstruction risk in patients with apical HCM.	No specific treatment provided
	TNNI3, TNNT2, TPM1	Hypertrophy in the anterior wall, interventricular septum, and lateral wall of the left ventricle.	Beta-blockers, ICDs
	TTN, OBSCN	Uniform hypertrophy across the myocardium.	Regular monitoring, medications, ICDs
Zhou et al. [10]	MYBPC3	LVMWT and have a higher LVMWT/LVPWT ratio.	Beta-blockers Septal myectomy in severe cases​
	MYH7	LVMWT and have a higher LVMWT/LVPWT ratio	Beta-blockers, Septal myectomy in severe cases​
Zhang et al. [11]	MYBPC3	LVWT and a higher prevalence of late gadolinium enhancement	Beta-blockers, Calcium channel blockers, or Septal myectomy
Wu et al. [12]	FHOD3	linked to a higher risk of cardiovascular death and SCD.	No specific treatment provided
Wasserstrum et al. [13]	MYBPC3	Variable clinical presentation: asymptomatic cases, severe hypertrophy with a higher risk of HF and arrhythmias.	Beta-blockers, Calcium channel blockers, or ICDs
	MYH7	Left ventricular hypertrophy, often involving the septum.	Beta-blockers, Calcium channel blockers, ICDs
Velicki et al. [14]	MYBPC3	Dyspnea, mitral leaflet abnormalities and systolic anterior motion.	No specific treatment provided
	MYH7	Linked to more severe forms of HCM: palpitations, mitral leaflet abnormalities, systolic anterior motion, and mitral annulus calcifications.	No specific treatment provided
Trachoo et al. [15]	MYBPC3	Associated with HCM, LVH, shortness of breath, chest pain, palpitations	Beta-blockers, Calcium channel blockers Regular monitoring
	MYH7	Severe HCM	Beta-blockers, Calcium channel blockers, ICDs.
	TTN	Associated with DCM, LVD,systolic dysfunction, HF, and arrhythmias.	ACE inhibitors, Beta-blockers, Diuretics ICDs or CRT
	SCN5A	associated with arrhythmias, DCM.	No specific treatment provided
Stava et al. [16]	MYBPC3	associated with HCM, contributing to left ventricular hypertrophy.	Beta-blockers, Calcium channel blockers, ICDs
	MYH7	associated with HCM, often involving significant ventricular thickening.	Beta-blockers, Calcium channel blockers, ICDs
	TTN	DCM, linked to LVD and systolic dysfunction, HF and arrhythmias.	ACE inhibitors, Beta-blockers, diuretics, ICDs or CRT,
	PKP2	ARVC	Beta-blockers, ICDs, Lifestyle modifications, including avoidance of strenuous exercise.
Ramensky et al. [17]	MYBPC3	HCM, increased risk of SCD	Beta-blockers, ICDs, Regular monitoring.
	MYH7	HCM	Beta-blockers, ICDs, regular monitoring.
	DSP	ARVC, arrhythmias and HF.	Antiarrhythmic medications, lifestyle modifications, ICDs
	KCNQ1	long QT syndrome, increased risk of ventricular arrhythmias and SCD.	Beta-blockers
Nakashima et al. [18]	MYBPC3	HCM, LVH, HF and arrhythmic events.	ICDs, Regular follow-up and genetic counseling
	MYH7	severe forms of HCM: NSVT, associated with a higher risk of SCD and other arrhythmic events.	ICDs, Regular follow-up and genetic counseling
	TNNT2	Associated with HCM, particularly with an increased risk of arrhythmic events, including ventricular tachycardia and fibrillation.	No specific treatment provided
	TNNI3	Associated with HCM, particularly with an increased risk of arrhythmic events, including ventricular tachycardia and fibrillation.	No specific treatment provided
	TPM1	associated with significant hypertrophy and arrhythmic events	No specific treatment provided
Morita et al. [19]	MYBPC3	LVH, shortness of breath, chest pain, and an increased risk of SCD.	Beta-Blockers, Calcium Channel Blockers, ICDs, Lifestyle Modifications.
	MYH7	severe HCM, ventricular arrhythmias, increased risk of SCD.	Beta-Blockers Calcium Channel Blockers Antiarrhythmic Medications, ICDs, Lifestyle Modifications.
	TNNT2	Associated with an increased risk of arrhythmic events, including ventricular tachycardia and fibrillation, higher risk of SCD	No specific treatment provided
Moriki et al. [20]	MYH7	Associated with severe forms of HCM, characterized by significant LVH, higher risk of SCD	Beta-block ICDs Genetic counseling
	MYBPC3	Milder form of HCM, with a later onset of symptoms and a slower progression of the disease.	Beta-blockers Calcium channel ICDs
	TNNT2	Linked to a higher risk of SCD, mild LVH. It is often associated with exercise-induced arrhythmias.	Beta-block ICDs Genetic counseling
	TNNT3	severe HCM, high risk of SCD.	Beta-blockers ICDs Regular follow-up
Meshkov et al. [21]	MYH7	Associated with LVNC (dilated LVNC and hypertrophic LVNC) and other cardiomyopathies.	Beta-blockers ICDs
	TTN	Correlates with worse cardiac function and a higher likelihood of developing arrhythmias.	Beta-blockers ACE inhibitors ICDs
	ACTN2	LVNC, hypertrophic and dilated forms of LVNC.	No specific treatment provided
	SLC22A5	associated with primary carnitine deficiency, LVNC, DCM	Supplementation with carnitine and management of HF symptoms. Genetic counseling
	VCL	associated with LVNC and other forms of cardiomyopathy, HF and arrhythmias.	ICDs Regular cardiac monitoring
	FHOD3	hypertrophic and dilated forms of LVNC	Medications for HF and arrhythmias.
McGurk et al. [22]	MYH7	HF and arrhythmias.	Beta-blockers ACE inhibitors ICDs
	MYBPC3	Associated with late-onset and milder forms HCM.	ICDs
	TNNT2	HCM and DCM, often with a higher risk of SCD	beta-blockers and ICDs
Mazzaccara et al. [23]	MYH7	Strongly associated with HCM and DCM	Beta-blockers ICDs
Mattivi et al. [24]	MYH7	significant LVH	Beta-blockers
	MYBPC3	HCM with risks of SCD and HF. The degree of hypertrophy can vary widely.	Beta-blockers Calcium channel blockers ICDs
Magrì et al. [25]	MYBPC3	HCM, associated with a later onset of disease and a milder phenotype, risk of HF and SCD.	Beta-blockers Calcium channel blockers. ICD
	MYH7	HCM, significant LVH and a higher risk of SCD.	Beta-blockers Calcium channel blockers. ICDs
Lorenzini et al. [26]	MYBPC3	associated with a range of phenotypes from mild to severe HCM, significant risk of SCD.	Beta-blockers, Calcium channel blockers
Lakdawala et al. [27]	MYBPC3	HCM, LVH and increased risk of HF and SCD.	Beta-blockers, Calcium channel blockers, ICDs
	MYH7	HCM and are often associated with significant LVH, high risk for SCD.	Beta-blockers, ICDs Regular monitoring Genetic counseling
Lacaze et al. [28]	MYBPC3	LVH, arrhythmias, and an increased risk of SCD.	Beta-blockers, Calcium channel blockers
	MYH7	LVH and are linked to a higher risk of SCD.	Beta-blockers Calcium channel blockers
Huurman et al. [29]	MYBPC3	HCM, HF and increased risk of SCD.	Beta-blockers Calcium channel blockers
	MYH7	HCM and a higher risk of SCD.	Beta-blockers ICDs
Huurman et al. [30]	MYBPC3	modest HCM and abnormal ECG findings.	No specific treatment
	MYH7	associated with significant hypertrophy	Regular monitoring and intervention when necessary.
	MYL2	milder phenotypes but still present a risk of developing HCM.	Close monitoring and medical management.
Hughes et al. [31]	MYBPC3	Microvascular dysfunction and myocardial perfusion defects in mutation carriers without overt hypertrophy.	No specific gene-targeted treatment was identified.
	MYH7	significant perfusion defects	No specific gene-targeted therapy
	TNNI3	microvascular abnormalities and perfusion defects.	Therapies, such a diltiazem and Valsartan, are under investigation for mutation carriers without hypertrophy.
Huang et al. [32]	MYH7	A higher degree of myocardial fibrosis.	Beta-blockers Calcium channel blockers.
	MYBPC3	milder myocardial fibrosis.	Beta-blockers and monitoring.
Holliday et al. [33]	MYBPC3	HCM: Three key variants were found: ○ c.1224-52G>A: Creates a new splice acceptor site, leading to a 50 bp extension of exon 14. ○ c.1090+453C>T: Activates a 77 bp cryptic exon between exons 12 and 13. ○ c.1928-569G>T: Causes cryptic exon splicing in intron 20.	The study explored the use of AOs to inhibit the aberrant splicing. In one case, AOs targeting exon 12a successfully restored up to 97% of the normal MYBPC3 transcript.
Heliö et al. [34]	MYBPC3	HCM, LVH, diastolic dysfunction, HF symptoms, and an increased risk of SCD.	Beta-blockers, Calcium channel blockers, ICDs
	MYH7	Severe hypertrophy and an early onset of symptoms, ventricular arrhythmias.	Beta-blockers Calcium channel blockers
	TNNT2	Relatively mild hypertrophic phenotype but carry a higher risk of arrhythmias and SCD.	No specific gene-targeted therapy
	TNNI3	hypertrophy and an increased risk of arrhythmias.	Risk stratification and management focus on preventing SCD, with ICDs
Hathaway et al. [35]	MYBPC3	HCM, associated with severe hypertrophy and increased risk of arrhythmias.	No specific gene-targeted therapy
	MYH7	Associated with severe LVH and a higher risk of SCD.	No specific gene-targeted therapy.
	TPM1	moderate to severe hypertrophy with variable penetrance.	Beta-blockers ICDs
	TNNI3	Associated with mild hypertrophy but increased risk of sudden cardiac death in some cases.	ICD implantation when appropriate.
	JPH2	Specific missense mutation (c.482C>A; p. Thr161Lys), causing hypertrophy.	No specific gene-targeted therapy .
	TNNT2	Associated with less severe hypertrophy but a high risk of sudden death.	Lifestyle modifications ICD implantation.
Earle et al. [36]	MYBPC3	Severe LVH and are at a higher risk for SCD and HF.	Beta-blockers, Lifestyle modifications ICDs.
	MYH7	Significant LVH and are associated with a younger age of onset of symptoms.	No specific gene-targeted therapy
	TNNT2	HCM: splicing defects, leading to in-frame deletions.	No specific gene-targeted therapy
de la Rosa et al. [37]	MYBPC3	Associated with severe LVH, severe diastolic dysfunction and higher left atrial (LA) size.	Beta-blockers ICDs
	MYH7	Mutations in MYH7 often lead to early onset of severe hypertrophy, and patients are at higher risk for adverse outcomes such as SCD and HF.	Beta-blockers, Calcium channel blockers Regular monitoring
	TNNT2	Associated with a more severe hypertrophic phenotype and increased risk of arrhythmias and SCD.	Beta-blockers, Calcium channel blockers Regular monitoring
de Feria et al. [38]	Sarcomere Mutation Positive HCM: MYBPC3	Significant asymmetric LVH and a higher likelihood	Beta-blockers, Calcium channel blockers, ICDs
Dai et al. [39]	RBM20 Gene	associated with a higher risk of SCA, impaired left ventricular systolic function (reduced LVEF), and higher risk of SCD.	Beta-blockers, Calcium channel blockers Regular monitoring
Chumakova et al. [40]	MYBPC3	variant MYBPC3 p.Q1233* is associated with obstructive HCM.	Beta-blockers, ICDs, SRT in cases of severe obstruction.
	MYH7	Associated with asymmetric septal hypertrophy and an increased risk of arrhythmias.	beta-blockers, ICDs, and SRT when indicated.
	TPM1	Recurrent variant TPM1 p.Q210R and is associated with moderate LV hypertrophy without obstruction at rest.	ICD for high-risk patients.
	FLNC	FLNC variants were associated with more severe clinical manifestations, including higher rates of HF progression.	Advanced therapies such as ICD implantation.
Chen et al. [41]	MYBPC3	Significant LVH, associated with a higher risk of SCD and HF progression.	No specific treatment provided
	MYH7	severe forms of HCM with marked LVH and an elevated risk of arrhythmias.	Beta-blockers or Calcium channel blockers
Canepa et al. [42]	MYBPC3	Associated with severe LVH, HF and SCD.	No specific treatment provided
	MYH7	severe hypertrophy, especially asymmetric LVH.	Beta-blockers and calcium channel blockers.
Butzner et al. [43]	MYH7	Severe form of HCM, LVH, a higher risk of SCD, arrhythmias, and HF.	β-blockers Calcium channel blockers, ICD, Septal myectomy Alcohol septal ablation
	MYBPC3	associated with a milder form of HCM, LVH and varying degrees of outflow obstruction.	β-blockers Calcium channel blockers, ICD, Septal myectomy, Alcohol septal ablation
	TNNT2	mild or even absent hypertrophy, exercise-induced symptoms such as syncope or sudden cardiac arrest.	ICD: prophylactic ICD implantation, β-blockers or Calcium channel blockers
Butters et al. [44]	MYBPC3	HCM, associated with LVH	β-blockers Calcium channel blockers, ICD, Septal myectomy Alcohol septal ablation.
	MYH7	HCM, associated with severe hypertrophy and early onset of symptoms, LVH and a higher risk of arrhythmias and SCD.	β-blockers Calcium channel blockers, ICD, Septal myectomy or alcohol septal ablation.
	TNNT2	linked to a higher risk of SCD, often without severe hypertrophy.	β-blockers.
Boen et al. [45]	TTN (Titin)	DCM, often severe and requiring HTx.	No specific treatment provided
	MYBPC3	HCM) and LVNC. Often leads to HTx in severe cases.	No specific treatment provided
	RBM20	DCM, with severe manifestations such as HF leading to HTx.	No specific treatment provided
	DSP	Associated with ACM and DCM.	No specific treatment provided
	PRDM16	DCM, often presenting with additional factors like alcohol abuse.	No specific treatment provided
Arabadjian et al. [46]	MYH7	HCM with significant LVH, including mid-ventricular and sub-basal hypertrophy.	ICD, Beta-blockers, Septal myectomy
	MYBPC3	HCM, often presents with basal or sub-basal hypertrophy and may include significant fibrosis.	beta-blockers, calcium channel blockers, and ICD, myectomy may be necessary for severe outflow tract obstruction.
	TNNI3	milder hypertrophy and a variable risk of arrhythmias.	Monitoring for arrhythmias and ICD implantation in high-risk cases.
	MYL3	hypertrophy, though the degree of clinical severity varies. Patients may be asymptomatic or have a moderate risk of arrhythmias.	beta-blockers and ICDs for those with risk factors for sudden death.
Antoniutti et al. [47]	MYH7 Gene (p.Arg652Lys Variant)	○ HCM in 64% of carriers ○ asymmetric septal hypertrophy in 75% of patients ○ Some patients exhibit LVOT obstruction (28%). ○ sudden death and embolic events. ○ NSVT was found in 8% of the patients. ○ AF occurred in 12% of the carriers. ○ There was incomplete penetrance, meaning not all carriers developed HCM.	○ Risk Stratification: Sudden cardiac death risk is assessed, and in high-risk patients, ICDs are recommended to prevent SCD. ○ Medical Therapy: Symptomatic patients receive beta-blockers or calcium channel blockers to manage symptoms like LVOT obstruction. ○ Surgical myectomy ○ Genetic Counseling.
Amr et al. [48]	MYBPC3	HCM, LVH, milder form of HCM but are at risk for SCD.	Genetic testing, cardiac monitoring, beta-blockers, ICD in high-risk individuals.
	MYH7	severe forms of HCM, arrhythmias and SCD, LVH and obstructive forms of the disease.	Risk stratification is crucial, ICD for high-risk patients, beta-blockers or calcium channel blockers.
	TNNT2	Associated with a higher risk of SCD, even in patients with less severe hypertrophy.	ICD implantation due to the high risk of fatal arrhythmias, even in the absence of significant LVH.
	TNNI3	Arrhythmias, associated with a higher risk of SCD, even in patients with less severe hypertrophy.	Risk stratification, including genetic findings, is essential for deciding on ICD implantation.
Allouba et al. [49]	MYBPC3	HCM. Homozygous truncating variants were associated with severe early-onset HCM, often diagnosed in childhood.	Genetic testing helps in early diagnosis and monitoring. ICDs may be recommended.
	MYH7	HCM: variants like the truncating mutation c.5769delG are associated with severe phenotypes.	beta-blockers, calcium channel blockers, ICDs for SCD prevention in high-risk individuals.
	MYL2	More severe HCM phenotypes, including greater wall thickness.	Require aggressive management, including early genetic testing for family members and consideration of ICDs depending on their risk profile.
	MYL3	More severe HCM phenotypes, including greater wall thickness.	ICD placement may be necessary, particularly in patients with severe hypertrophy and a family history of SCD.
	TRIM63	HCM	Family screening is essential, and affected individuals require careful monitoring due to the potential for severe HCM progression.
	CSRP3	Homozygous truncating variants are associated with milder adult-onset HCM	Regular monitoring of cardiac function, ICD implantation for high-risk individuals.
Afana et al. [50]	PLN	○ HCM, DCM, and ACM. ○ septal hypertrophy ○ Pathological Q waves and NSVT	○ ICD for high-risk of arrhythmias and SCD. ○ Standard HF treatments and anti-arrhythmic medications, and cardiac monitoring.
	MYBPC3	○ HCM, usually associated with asymmetric septal hypertrophy. ○ May develop progressive HF.	○ Beta-blockers, calcium channel blockers. ○ ICD for high risk of SCD.
	MYH7	○ severe forms of HCM, arrhythmias, SCD. ○ Septal hypertrophy, LVOT obstruction.	○ beta-blockers and calcium channel blockers. ○ ICD for SCD prevention in high-risk patients.
	TTN	○ DCM, LVD and systolic dysfunction. ○ HF	○ beta-blockers and ACE inhibitors. ○ In severe cases, HTx is required.
